# Bilateral Strength Deficit Is Not Neural in Origin; Rather Due to Dynamometer Mechanical Configuration

**DOI:** 10.1371/journal.pone.0145077

**Published:** 2015-12-18

**Authors:** Emilie Simoneau-Buessinger, Sébastien Leteneur, Anis Toumi, Alexandra Dessurne, François Gabrielli, Franck Barbier, Jennifer M. Jakobi

**Affiliations:** 1 Laboratoire d'Automatique, de Mécanique et d'Informatique industrielles et Humaines (LAMIH)–UMR CNRS 8201, Université de Valenciennes et du Hainaut-Cambrésis (UVHC), Valenciennes, France; 2 Laboratoire de l’Effort et du Mouvement, Haute Ecole Provinciale de Hainaut (HEPH)—Condorcet, Tournai, Belgium; 3 School of Health and Exercise Sciences, University of British Columbia Okanagan, Kelowna, Canada; Semmelweis University, HUNGARY

## Abstract

During maximal contractions, the sum of forces exerted by homonymous muscles unilaterally is typically higher than the sum of forces exerted by the same muscles bilaterally. However, the underlying mechanism(s) of this phenomenon, which is known as the bilateral strength deficit, remain equivocal. One potential factor that has received minimal attention is the contribution of body adjustments to bilateral and unilateral force production. The purpose of this study was to evaluate the plantar-flexors in an innovative dynamometer that permitted the influence of torque from body adjustments to be adapted. Participants were identically positioned between two setup configurations where torques generated from body adjustments were included within the net ankle torque (locked-unit) or independent of the ankle (open-unit). Twenty healthy adult males performed unilateral and bilateral maximal voluntary isometric plantar-flexion contractions using the dynamometer in the open and locked-unit mechanical configurations. While there was a significant bilateral strength deficit in the locked-unit (p = 0.01), it was not evident in the open-unit (p = 0.07). In the locked-unit, unilateral torque was greater than in the open-unit (p<0.001) and this was due to an additional torque from the body since the electromyographic activity of the agonist muscles did not differ between the two setups (p>0.05). This study revealed that the mechanical configuration of the dynamometer and then the body adjustments caused the observation of a bilateral strength deficit.

## Introduction

Experimental reports consistently reveal that activity on one side of the body affects the opposite side. It is also stated with little controversy that the underlying site of distinction between one and two limbs in movement execution and control is neural [1,2]. However, the results of the abundance of studies that center on maximal force production when two homologous muscles are simultaneously active (bilateral) compared with the summed force of homologous muscles contracting alone (unilateral) are contentious [3]. Early reports suggested that maximal bilateral contractions yielded lower force than the unilateral condition and this phenomenon became studied as the bilateral strength deficit [4,5]. Albeit with controversy, the bilateral strength deficit has been observed in both upper and lower limbs, across a number of contraction types (dynamic, isometric) and muscle groups but the extent and significance of the effect ranges considerably [3]. For example, the bilateral strength deficit is greater in the lower limbs and in dynamic contractions than in the upper limbs and in isometric contractions, respectively [6–11]. Overall, the inconsistency in which the bilateral strength deficit is reported [3,12–14] contributes to the underlying mechanism(s) remaining equivocal.

The foremost theory presented in the literature centers around neural inhibition during bilateral contractions. Spinal reflex activity associated with motor neuron excitability has gathered substantial interest with strong deliberation around the type II motor neurons being most affected due to the maximal nature of the contractile efforts [3,15,16]. More recently, investigations have also suggested that the bilateral strength deficit is due to interhemispheric inhibition of the motor cortex [17]. Evidence was also presented demonstrating that activation in the precentral gyrus decreased during bilateral contractions, which suggested that the cause of the bilateral strength deficit was upstream of the motor cortex [10]. Despite these efforts that give attention to the neural origin, the robustness of this phenomenon is weak across studies and thus the underlying cause of the bilateral strength deficit cannot be clearly distinguished as a neural source [13,14].

Additional to the neural approach, the concept of postural stability and body mechanics varying between unilateral and bilateral contractions was suggested [3,9,18]. Generally, multijoint complex movements (lateral pull-down, leg press) have exhibited a greater bilateral strength deficit than single-joint simple movements (*eg*, knee extension) [3,9,19] because they involve larger muscles and higher forces, thus the stability requirements to maintain position would be greater [20] and deter from the intended force output and in-turn the bilateral strength deficit would manifest. This theory was initially proposed through the study of simultaneous non-homonymous muscle activity [18] and reviewed across studies [3]. Recently, the postural theory was further explored by comparing upper body hand exercise with lower body leg extension [9,21]. However, this compelling argument for postural contributions is lessened because spinal connections are disparate between the upper and lower body [22], postural adjustments vary between sitting and standing [23,24], and the magnitude of force production is greater in leg extension compared with hand grip. No study has directly assessed the contribution of postural adjustments to the bilateral strength deficit through an evaluation of one joint and contraction type. In fact, the term ‘postural adjustments’, as used in the above-mentioned studies, refers to the action of muscles that are implied in postural stability.

**Fig 1:**
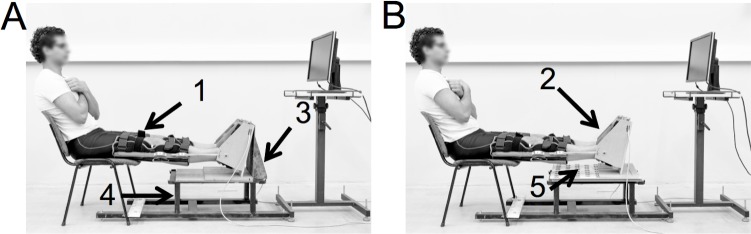
The ‘Booted, Open-Unit, Three dimension, Transportable, Ergometer’ (B.O.T.T.E.). (A) The B.O.T.T.E. in a locked-unit configuration. An orthosis was placed tight around the lower limb with the knee in full extension [1], the foot was secured into the footplate [2] which was attached to a block placed vertically behind the footplate [3] and this unit was locked to the base of the platform [4] which prevented movement in the locked-unit configuration. (B) The B.O.T.T.E. in an open-unit configuration with the ball-bearing housing [5] placed under the heel of the footplate. This allowed movement of the lower limb horizontally but kept the knee and ankle joints in the established positions within the orthosis. The force-torque sensor is situated under a plate on which is placed the sole of the foot. Please see [25] for more details. The individual in this picture has given written informed consent (as outlined in PLOS consent form) to publish this image.

Another way to explain the bilateral strength deficit could be the ‘body adjustments’ while positioned in the dynamometer. Indeed, the common point of the previous studies that observed a bilateral strength deficit is the use of dynamometers that allow body counterbalances. In this manner, torques/forces generated in other parts of the body can contaminate the examined joint torque output. To achieve a direct measure of the contribution of body adjustments to net unilateral and bilateral torque outputs, a customized isometric plantar-flexion (PF) dynamometer was designed [25]. This innovative ‘Booted, Open-Unit, Three dimension, Transportable, Ergometer’ (B.O.T.T.E.) allowed net torque to be localized to the ankle (open-unit) as well as inclusive of body adjustments (locked-unit) through two configurations of setup. Within the locked-unit, the back of the chair and the base of the boot dynamometer were linked within a singular framework and this constrained the body within the testing device and coupled the contribution of forces from body adjustments to the net torque at the ankle (Fig 1A). In the open unit configuration, the ankle, leg, knee, hips and body were identically positioned, but the removal of the reinforcing plate from behind the ankle apparatus as well as placement of a ball-bearing element under the ‘boot’ caused the body to become uncoupled with the setup and torque was localized to PF of the ankle (Fig 1B). Thus, the purpose of the study was to evaluate one muscle group in one singular type of contraction undertaken in one common position to determine whether body adjustments contribute to the bilateral strength deficit. It was hypothesized that for PF there would be a bilateral strength deficit in the locked-unit but not in the open-unit. Our assumption was during maximal PF in the locked-unit the trunk would transmit weight to the back of the chair, and since the leg is fixed, muscular actions at any one joint in the kinetic chain would be additive to the torque recorded at the ankle. This phenomenon would be magnified in the unilateral condition when the upper body counterbalances the single contracting leg by torsion around the hips and this would also generate an additional torque. Because body adjustments are ineffective in the open-unit, only ankle torque from the PF contraction would contribute and this would result in similar torque in bilateral and summed unilateral contractions.

## Materials and Methods

### Participants

This study was conducted on 20 healthy adult males (age: 22.7 ± 2.9 years, body mass: 72.7 ± 15.1 kg, height: 1.76 ± 0.07 m). Nineteen of the participants self-identified as right leg dominant [26]. All volunteers gave their written consent prior to participation and all procedures complied with the Declaration of Helsinki and were approved by the Ethics Committee of the University of Valenciennes. None of the participants had any major or recent musculoskeletal injury in the legs.

### Mechanical recordings

An ankle dynamometer ‘Booted, Open-unit device, Three dimension sensor, Transportable, Ergometer (B.O.T.T.E.)’ was developed to record isometric torque at the ankle (Fig 1). To assess torque in both the right and left legs two identical dynamometers were simultaneously used. This innovative ankle dynamometer is based on a boot design and it allows evaluation of torque in a locked- as well as open-unit configuration. Toumi et al. [25] described the mechanical system of this device, validated it and fully explained how ankle torque calculations are made. Briefly, it is composed of an adjustable orthosis to maintain the knee in extension and a footplate mounted with a 6-component force-torque sensor (Sensix, France). The force-torque sensor is situated under a plate on which is placed the sole of the foot. The multi-axis load cell measurements (forces, torques) were used for the tridimensional ankle torque calculation in the locked- and open-unit configurations [25].

In both the open- and closed-unit configurations the participant was seated with the hips at ~60° with the legs extended straight forward and the ankle joints placed at 10° of PF. An orthosis was fastened from approximately mid-shank to mid-thigh on each leg to ensure the legs were extended forward for the duration of the experiment. The participant’s arms were folded across the chest during testing. The axis of rotation of the ankle joint (*i*.*e*. the bimalleolar axis) was aligned with the axis of rotation of the dynamometer. Each foot was held in this position and within the dynamometer by a soft orthosis that was tightly attached to the footplate through custom straps at the heel and around the ankle joint. An additional strap was placed over the metatarsophalangeal joints to maintain and standardize forefoot position.

Testing in the locked-unit occurred by fastening the boot of the dynamometer to the platform by a block that was placed vertically at the base of the boot (Fig 1A). The open-unit configuration was created by removing the block to unlock the boot from the platform and by placing the heel of the boot onto a ball-bearing housing. This allowed movement of the lower limb horizontally but kept the knee and ankle joints in the established positions within the orthosis (Fig 1B). Torque was sampled at 2 kHz using a custom built Matlab program (Matlab 7.12, The MathWorks Inc., MA, USA) and a 16-bit A/D board (National Instruments, Texas, USA) interfaced to a personal computer.

### EMG recordings

An 8-channel wireless EMG system (Aurion Inc., Italy) was used to record EMG from the soleus, gastrocnemius medialis, gastrocnemius lateralis and tibialis anterior muscles of both legs. Two silver-chloride surface electrodes of 10-mm diameter (Controle Graphique Medical, Brie-Comte-Robert, France) with an inter-electrode distance of 25 mm were placed on each muscle bilaterally according to the European Recommendations for Surface ElectroMyoGraphy [27]. To reduce impedance at the skin-electrode interface, the skin was exfoliated with shaving and gentle abrasion and cleaned with an alcohol-based tissue pad. The EMG signals were sampled at 2 kHz, and each channel had a gain of 1000x with a bandpass filter of 10–400 Hz.

### Neuromuscular electrical stimulation procedure

A stimulator (Combi 500, GymnaUniphy, Belgium) was used to deliver biphasic symmetric rectangular pulses of 75 Hz and 400 μs duration [28] with an on to off ratio of 5s:10s. A stimulating pad (10 x 5 cm) was placed approximately 3 cm distal to the popliteal fossa and the second stimulating electrode was positioned over both the medial and lateral gastrocnemius muscle bellies, along the midline. The position of the second stimulating electrode on the gastrocnemii was superior to the soleus EMG electrodes and inferior to the gastrocnemii EMG electrodes.

### Experimental protocol

Participants visited the laboratory once. The experimental session comprised maximal voluntary isometric contractions (MVC) and electrically evoked contractions for PF, with concomitant EMG and torque recordings. Dorsiflexion MVC was also assessed to normalize the tibialis anterior EMG for quantification of coactivation. All the contractions were performed under isometric conditions using separate B.O.T.T.E. dynamometers for the left and right legs. Prior to testing with each type of setup configuration (open- and locked-unit), each participant performed a standardized warm-up/familiarization consisting of several submaximal contractions of increasing intensity in both dorsiflexion and PF to become accustomed to the test procedure.

Each participant was initially familiarized with several submaximal electrical stimuli. The stimulation intensity was progressively increased until maximal evoked torque and/or maximum tolerated level of stimulation were achieved. Only the participants who showed no increase in twitch torque with an increase in stimulation intensity were included (5 out of 20 were excluded). This was done for both legs consecutively. Once the supramaximal intensities were determined, they were then maintained for the entire session of electrostimulation. The contraction type (MVC and electrostimulation), the task (unilateral right, unilateral left and bilateral) as well as the type of setup configuration (open- and locked-unit) were randomized.

The participants performed at least two ~5-s step MVC per testing configuration (open-unit; locked-unit). If the two MVC were not within 5%, another trial was executed. To motivate the participants to maximally activate their muscles, they were strongly encouraged by investigators and a real-time visual feedback of the torque was provided [29]. A resting time of at least 2-min was taken between each trial to avoid any effect of fatigue on the MVC measurements. For the electrostimulation condition three maximal tetanic contractions were induced.

### Data analysis

The torque was calculated as described in Toumi et al. [25]. The force-torque signal was filtered by a 10 Hz low-pass filter (2^nd^ order Butterworth filter, zero lag) and was corrected for gravity by subtracting the baseline. The MVC and electrostimulation trials resulting in the highest generated torque in each condition were used for further analysis. The maximal torque corresponded to the maximal mean torque reached during the contraction over a 0.5-s period.

For the locked- and open-unit configurations, the bilateral index was calculated for both voluntary and electrically-induced contractions using the maximal torques from unilateral left, unilateral right and bilateral contractions. The Bilateral Index (%) = (bilateral torque / (unilateral left torque + unilateral right torque))*100 was also determined and a score lower than 100% was indicative of a bilateral strength deficit whereas a bilateral index higher than 100% suggested a bilateral facilitation [30]. EMG was quantified as root mean square amplitude over the 0.5-s period that corresponded to the maximal plateau of torque. To evaluate the level of coactivation, the EMG activity recorded from the tibialis anterior during PF was divided by the maximal EMG of the tibialis anterior recorded during dorsiflexion, and expressed as a percentage of MVC. All data processing was performed off-line using a custom built Matlab program (Matlab 7.12, The MathWorks Inc., Natick, MA, USA).

### Statistics

Data are reported as mean ± standard deviation (SD). The unilateral and bilateral left and right PF torques were used to compute the Bilateral Index (%) for the locked- and open-unit configuration in voluntary and electrically stimulated contractions. These indexes for voluntary and electrostimulation were independently evaluated with a test of means against the reference value of 100% to determine whether a deficit or facilitation was evident. Then, a paired t-test was also used to compare these indexes between the locked- and open-unit configurations. A task (unilateral, ‘separated’ bilateral) x setup configuration (locked-unit, open-unit) x side (left, right) 3-way repeated measures analysis of variance (ANOVA) was conducted for voluntary and electrically stimulated contractions. Agonist EMG of the triceps surae (gastrocnemius lateralis, gastrocnemius medialis and soleus muscles) as well as tibialis anterior EMG co-activation in the voluntary condition were analyzed with a 3-way ANOVA (task x setup configuration x side). In the occurrence of significant interactions, a Fisher Post hoc test was applied. Significance was accepted at p < 0.05. Where appropriate, Cohen’s d coefficient or partial eta-squared (η_p_²) was calculated for statistically significant parameters to estimate effect size.

## Results

**Fig 2:**
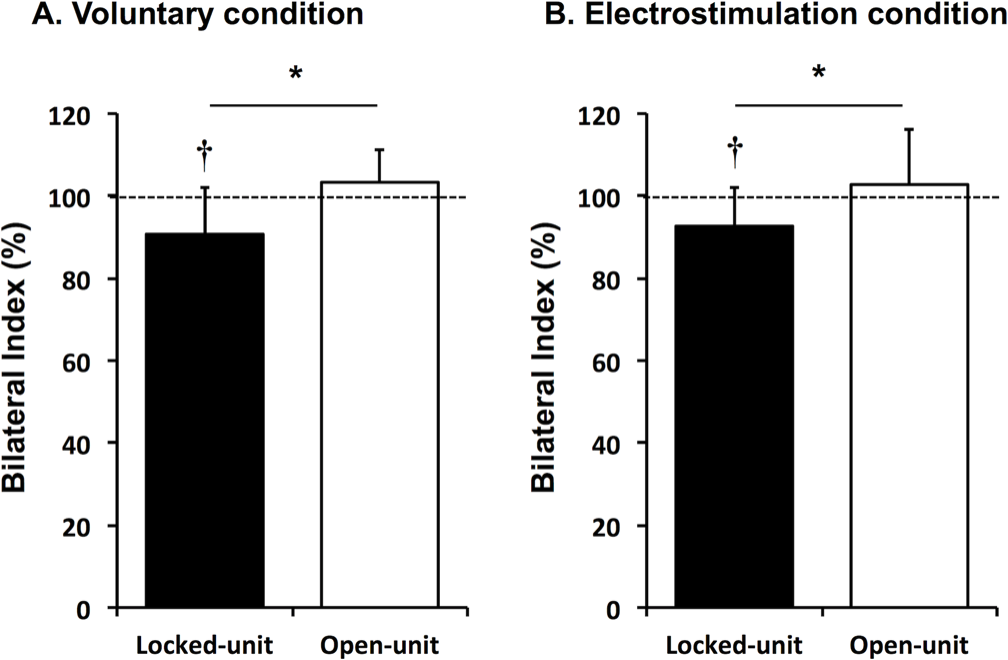
Bilateral Index for the locked-unit (filled bar) and open-unit (open bar) setup configurations for voluntary (A) and electrical stimulation (B) conditions. † Significantly different from a Bilateral Index of 100% (p<0.01). * Significant difference between setup configurations (p<0.01).

During maximal voluntary contractions, the Bilateral Index for the locked-unit configuration (90.7 ± 11.4%) was significantly lower than 100% (p<0.01) but it did not differ from 100% for the open-unit configuration (103.2 ± 7.8%; p = 0.08) (Fig 2A). The Bilateral Index for the locked-unit was also significantly lower than the one for the open-unit for the voluntary condition (p = 0.001, d = 1.09). In the electrostimulation condition, the Bilateral Index was also significantly lower than 100% for the locked-unit configuration (92.6 ± 9.5%, p = 0.01) but the Bilateral Index for the open-unit also did not differ from 100% (102.7 ± 13.5%, p = 0.45) (Fig 2B). Similar to the voluntary contractions, in the electrostimulation condition the Bilateral Index for the locked-unit was lower than for the open-unit configuration (p = 0.01, d = 0.80).

**Fig 3:**
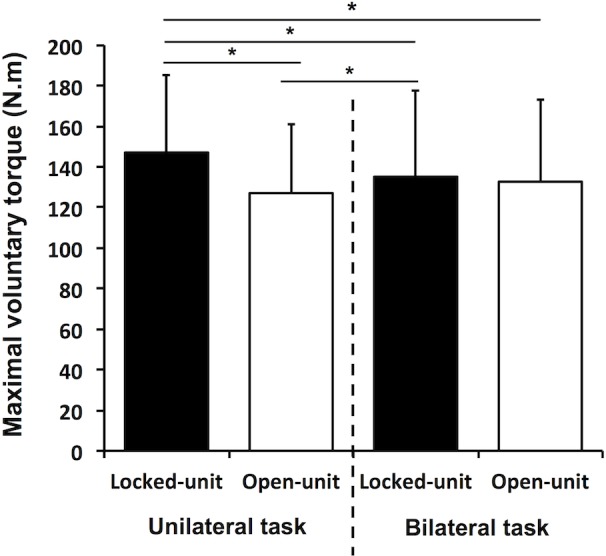
Maximal voluntary torque for the locked-unit (filled bars) and open-unit (open bars) in the unilateral and ‘separated’ bilateral tasks. * Significant difference between conditions (p<0.05).

There was a significant effect of task x setup configuration (F_1,19_ = 15.95, p<0.001, η_p_² = 0.46) in which the unilateral torque in the locked-unit configuration was significantly greater than the torques recorded in the other conditions (p<0.01) and the bilateral torque in the locked-unit configuration was higher than the unilateral torque in the open-unit configuration (p = 0.02) (Fig 3). Main effects revealed that the torque in the locked-unit configuration (141 ± 41 N.m) was greater than the one in the open-unit configuration (130 ± 37 N.m) (F_1,19_ = 10.68, p<0.01, η_p_² = 0.36) and the torque in the right leg (144 ± 41 N.m) was stronger than the torque in the left leg (127 ± 36 N.m) (p<0.001). For electrically induced torque, main effects revealed that the torque in the locked-unit configuration (60 ± 27 N.m) was greater than the one in the open-unit configuration (53 ± 27 N.m) (F_1,14_ = 20.84, p<0.001, η_p_² = 0.60).

There were no significant interactions for the analysis of EMG for agonist muscle activation (p>0.05). The right leg (0.24 ± 0.08 mV) had moderately higher levels of EMG agonist activity than the left leg (0.22 ± 0.07 mV) (F_1,19_ = 5.51, p = 0.03, η_p_² = 0.22). The repeated measures ANOVA for tibialis anterior coactivation revealed a significant interaction of task x setup configuration (F_1,19_ = 6.03, p = 0.02, η_p_² = 0.24). Coactivation in the locked-unit configuration was significantly higher in the unilateral task than in the bilateral task (15.6 ± 6.8% *vs* 14.8 ± 6.8%; p = 0.04) and the coactivation in the unilateral task was greater in the locked-unit than in the open-unit configuration (15.6 ± 6.8% *vs* 14.6 ± 6.7%; p = 0.02).

## Discussion

The principal finding of this study is that the supposed physiological phenomenon of the bilateral strength deficit is in fact due to the addition of torque from body adjustments. This study revealed that the mechanical configuration of the dynamometer might account for differences in unilateral and bilateral tasks in previous reports of bilateral strength deficit. Specifically, when using classical closed-unit configuration dynamometers, torques/forces generated in other parts of the body contaminate the ankle torque measurement. In the present study, the influence of torque from body adjustments was assessed at the ankle with an innovative dynamometer that allowed the participant to be identically positioned between setups where torque generated from body adjustments were included within the net ankle torque (locked-unit) or independent of the ankle (open-unit). The primary hypothesis was confirmed that when body adjustments were included within net torque there is a bilateral strength deficit. Yet, when measurements are localized to the joint of interest through the open-unit setup where additional torque from the body cannot be included, summed unilateral and bilateral torques were similar. These data of the mechanical contribution of torque to the bilateral strength deficit are further substantiated by the observation that agonist EMG did not differ between conditions, and unilateral torque was greater in the locked-unit than in the open-unit configuration. This unilateral difference between configurations supports the second hypothesis that trunk torsion to the contralateral side of the contracting single limb contributes force thereby generating an advantage in the unilateral condition over the bilateral condition. Overall, these data suggest that the bilateral strength deficit is not neural in origin, rather due to body adjustments allowed by the dynamometer.

Although there is clear evidence to indicate that the underlying cause of differences between bilateral and unilateral reaching, grasping and reaction times are mediated neurally [10] the association of the bilateral strength deficit with neural adaptations remains equivocal [3,31]. This study supports prior work that suggested the origin of the bilateral strength deficit was not neural [13,14]. The EMG agonist data for voluntary activation did not differ between the bilateral and unilateral contractions. Moreover, when neural drive was removed through the use of electrostimulation, the same outcome as for voluntary conditions was evident. In situations when bilateral strength deficits have been shown to be caused by neural mechanisms, the bilateral strength deficit in the lower limbs is typically greater than 7% [6–8]. But, when force is localized to the joint of interest and adjustments in body position are negligible within the net torque it seems that force and muscle activity differ minimally [9]. Controversy will likely remain surrounding the neural underpinnings of the bilateral strength deficit until the observation of the phenomenon is consistent.

In the locked-unit, when body adjustments contribute to the net torque, a bilateral strength deficit was evident, yet when torque was localized to the ankle joint in the open-unit and body adjustments were not contained within the net torque, the bilateral strength deficit was nonexistent. Thus, a comparative between these setups in-which participant position was identical across conditions, where the only change was the contribution of the body, distinguishes that when body adjustments do not contribute to the net torque there is no bilateral strength deficit for PF. These findings are consistent with those of Kawakami et al. [16] who have tested bilateral strength deficit in plantar-flexion in 2 leg positions: they actually showed that bilateral strength deficit was greater when the knee was extended than when bent to 90° (13.9% *vs* 6.6%). Indeed even if these authors did not explain their results in this way, one potential explanation for their findings is that it is possible to use body adjustments when the leg is extended but not when flexed thereby artificially increasing the PF torque. Contrary to this finding, a decline in the bilateral strength deficit was observed with training [11,19] due to neural adaptations [32]. However, these studies have not ascertained whether the training protocol induced postural adaptations that permitted the body to become more stabilized and in-turn produce greater force in the bilateral contractions. Overall, the potential for body adjustments to reduce the purported bilateral strength deficit with training remains unexplored.

The neuromuscular system is a particularly dynamic system in which force output is known to be modulated by task [33]. It is well understood that a perturbation applied to one segment of the body causes motion of adjacent segments through contact between joints [34,35]. Van der Fits et al. [24] also showed that isometric efforts are accompanied by dynamic body adjustments that are necessary to maintain equilibrium within the seated center of balance. This is in accordance with a variety of theories of postural reflex mechanisms that generate forces and increase the muscles recruited [36] in order to resist perturbations that deflect the position of the body from its initial posture [37] and these forces increase with the load of the task [24]. In the locked-unit configuration, the participant could move slightly at the hips and this would add weight to the back of the chair and increase the torque measured at the ankle joint. These body adjustments were transmitted, because the distal aspect (leg) is fixed and thereby muscular actions at any one joint in the kinetic chain would be additive to the torque recorded at the ankle in the locked-unit, but not within the open-unit configuration.

Although a plethora of studies have questioned the sensitivity of a variety of devices to measure force and torque [38–41], no study to-date has accounted for this when attempting to define the underlying cause of the bilateral strength deficit. Body adjustments are load dependent, thus it might be suggested that the bilateral condition would gain the greatest benefit through the contraction of both legs; yet, in the bilateral condition, counterbalance contributions are not evident as they are in the unilateral condition. When unilateral and bilateral arm contractions were compared in a seated apparatus, torsional reaction forces were larger during the unilateral condition [24,42]. Thus, the mechanical advantage in the bilateral contraction in the locked-unit configuration is countered by substantial torsional forces that augment the net torque output in the unilateral condition. This is evident in the current experiment as the unilateral locked-unit configuration generated higher torques than the unilateral open-unit configuration and each leg in both of the bilateral configurations (open- and locked-unit). It was suggested that body adjustments are likely anticipatory mechanisms specific to the task constraints [43]. This feed-forward aspect of planning might indicate why recent studies have identified neural inhibition between cortices or higher level planning as the underlying cause of the bilateral strength deficit [7,10]. It is plausible that these studies were detecting higher level shaping towards a bilateral or unilateral body adjustment.

There is a prominent antagonist coactivation generated to help stabilize the ankle joint during body adjustments. This postural contribution coupled with concurrent activation potentiation, also referred to as remote voluntary contraction would increase tibialis anterior activation but would have minimal influence on the net agonist EMG activity [44]. It is suggested that contraction of muscles outside the joint of interest may increase maximal activation level. This augmentation in activity is commonly attributed to motor irradiation and/or to an increase in spinal excitability [45] and has been most notably studied through jaw clenching, Valsalva maneuver and hand gripping [44], but muscle activity from adjacent sites also interact with the primary movers [46,47]. It would be very surprising for plantar-flexor activity to increase dramatically through remote contractions since these are apt to be almost maximally activated by volition, especially when verbal encouragements and visual feedback of torque are provided [29]. On the contrary, the tibialis anterior, which should be engaged through postural strategies to stabilize the joint, may also experience enhanced activation through irradiation from the body adjustments. Muscle activity from the torso directed towards maintaining the centre of balance but unrelated to the movement may have a neural influence on coactivation of the dorsiflexors and further enhance joint stability.

In conclusion, the present data demonstrated that body adjustments contribute substantially to torque and that when these adjustments are contained within a measure of PF the bilateral strength deficit is recorded. However, when the measured torque is only due to the ankle joint muscles, bilateral and unilateral torques are similar. The vast majority of the literature on the bilateral strength deficit has clearly speculated and attempted to study a neural phenomenon. These data indicate that further exploration into a neural phenomenon underlying the bilateral strength deficit needs to account for body adjustments in the measurements of torque. Within this arrangement the subconscious aspect of body adjustments in maintaining the center of balance between bilateral and unilateral efforts is a viable avenue of further exploration.
